# Effects of the Insulted Neuronal Cells-Derived Extracellular Vesicles on the Survival of Umbilical Cord-Derived Mesenchymal Stem Cells following Cerebral Ischemia/Reperfusion Injury

**DOI:** 10.1155/2020/9768713

**Published:** 2020-07-16

**Authors:** Yan Huang, Zuo Liu, Fengbo Tan, Zhiping Hu, Ming Lu

**Affiliations:** ^1^Key Laboratory of Protein Chemistry and Developmental Biology of Ministry of Education, College of Life Sciences, Hunan Normal University, Changsha, Hunan 410081, China; ^2^Department of Neurosurgery, Second Affiliated Hospital of Hunan Normal University, Changsha, Hunan 410003, China; ^3^Hunan Provincial Key Laboratory of Neurorestoration, China; ^4^Department of Gastrointestinal Surgery, Xiangya Hospital, Central South University, Changsha, Hunan 410008, China; ^5^Department of Neurology, The Second Xiangya Hospital, Central South University, Changsha, Hunan 410011, China

## Abstract

Umbilical cord-derived mesenchymal stem cells (UC-MSCs) engraftment is a potential therapy for cerebral ischemic stroke. However, the harsh microenvironment induced by cerebral ischemia/reperfusion restricts the survival rate and therapeutic efficiency of the engrafted UC-MSCs. In this study, we explored whether small extracellular vesicles (EVs) derived from injured neuronal cells following exposure to cerebral ischemia/reperfusion insult affect the survival of transplanted UC-MSCs. To establish a simulation of cerebral ischemia/reperfusion microenvironment comprising engrafted UC-MSCs and neuronal cells, we cocultured EVs derived from injured N2A cells, caused by exposure to oxygen-glucose deprivation and reperfusion (OGD/R) insult, with UC-MSCs in a conditioned medium. Coculture of UC-MSCs with EVs exacerbated the OGD/R-induced apoptosis and oxidative stress. Suppression of EVs-release via knock-down of Rab27a effectively protected the UC-MSCs from OGD/R-induced insult. Moreover, hypoxia preconditioning not only elevated the survival of UC-MSCs but also improved the paracrine mechanism of injured N2A cells. Altogether, these results show that EVs from injured N2A cells exacerbates OGD/R-induced injury on transplanted UC-MSCs *in vitro*. Hypoxia preconditioning enhances the survival of the engrafted-UC-MSCs; hence, thus could be an effective approach for improving UC-MSCs therapy in ischemic stroke.

## 1. Introduction

Ischemic stroke is a common cause of neurological dysfunction, disability, and even death [1]. The severity of ischemic stroke depends on the extent of cerebral ischemia and the cerebral reperfusion process. The latter may cause detrimental conditions such as elevating oxidative stress leading to irreversible injury [2–4], inflammatory response, and cell death [5, 6]. Mesenchymal stem cells (MSCs) have emerged as a potential therapy for ischemic stroke owing to their immunoregulation effects, ability to restore and regenerate injured tissues [7–9]. Notably, UC-MSCs exhibit rapid proliferation and low tumorigenicity. They are therefore an ideal source of MSCs for ischemia stroke therapy [10–12]. However, direct engraftment of MSCs into the injured brain area does not always yield satisfactory repair [13]. This is because the low transplantation and survival rates of UC-MSCs limit their therapeutic efficacy [13]. Therefore, it is imperative to develop appropriate methods for improving the survival of engrafted UC-MSCs.

The paracrine process plays an essential role in the engraftation mechanism of MSCs, and EVs are the critical component of this paracrine process [14, 15]. MSCs-derived EVs can be used as a cell-free therapy for the repair and regeneration of injured nerve cells. This is because EVs can cross the blood-brain barrier [16–19]. There are at least three subfamilies of EVs, including microvesicles, exosomes, and apoptotic bodies [20]. The molecules released by exosomes maintain homeostasis at the cellular microenvironment by removing damaged cytoplasmic DNA from cells [21]. However, a recent study reported that EVs may promote neuroinflammation and affect neuronal survival under ischemic stress condition [20]. However, it is currently unknown whether EVs secreted from neuronal cells that have been injured by cerebral ischemia/reperfusion process affect the survival of engrafted UC-MSCs.

Studies show that oxygen concentration is an important parameter that drives differentiation, proliferation, and renewal of MSCs [22, 23]. Hypoxia preconditioning of MSCs enhances their cell viability, survival after transplantation, and biological functions [24–26]. On the same note, hypoxia preconditioning enhances the release of EVs from MSCs, which is beneficial to promote neural regeneration and restoration in ischemic tissues [27]. However, it is not known whether hypoxia preconditioning will promote the survival of engrafted UC-MSCs.

In this study, to establish a simulation of cerebral ischemia/reperfusion microenvironment comprising engrafted UC-MSCs and neuronal cells, we cocultured EVs derived from injured N2A cells due to OGD/R insult with UC-MSCs as described previously [28]. We then examined the effect of EVs derived from injured neuronal cells on the survival rate of engrafted UC-MSCs. Additionally, we explored whether hypoxia preconditioning could promote the survival of the engrafted UC-MSCs via the paracrine mechanism of the injured N2A cells.

## 2. Materials and Methods

### 2.1. Cell Culture and Hypoxia Preconditioning

This study was approved by the Ethics Committee of the Hunan Normal University. The health parents of newborns enrolled in this study provided informed consent to participate in this study (the donor ID is 10305521). We harvested human fresh umbilical cord samples from newborn babies as previously described [29]. Briefly, the harvested umbilical cord samples were immediately washed and rinsed with phosphate-buffered saline (PBS) containing penicillin-streptomycin (Invitrogen, Carlsbad, CA, USA) twice to remove the cord blood. We then dissected the cords to about 10 mm in size. The dissected cords were cultured in Dulbecco's modified Eagle's medium (DMEM; Invitrogen, Carlsbad, CA, USA) containing 10% fetal bovine serum (FBS; Invitrogen, Carlsbad, CA, USA) at 37°C and 5% CO_2_ condition. We replaced the culture medium every 3-4 days after plating. After that, we transferred the cells to a new cell flask for further proliferation. The 3rd passage cells were used in subsequent experiments. Flow cytometry was used to identify the surface markers of UC-MSCs. We found that CD105, CD90, and CD73 were upregulated in UC-MSCs, while CD34 and CD45 were downregulated. Hypoxia preconditioning was performed on the UC-MSCs. Briefly, the cells were cultured in a specific chamber (Billups Rothenberg, Inc., Del Mar, CA) with 1%O_2_, 94%N_2_, and 5% CO_2_ for 48 hours. Normoxia preconditioning of UC-MSCs was performed under 21% O_2_ combined with 5% CO_2_ for 48 hours [27].

We purchased the N2A cells from the Cell Storage Center of the Chinese Academy of Sciences (Shanghai, China). The N2A cell-line was cultured in DMEM (Invitrogen, Carlsbad, CA, USA) media containing 10% FBS (Invitrogen, Carlsbad, CA, USA) and 1% penicillin-streptomycin at 37°C and 5% CO_2_.

### 2.2. Cerebral Ischemia/Reperfusion Injury *In Vitro* Model

To mimic *in vivo* ischemia/reperfusion microenvironment, the OGD/R model was established as previously reported [30]. Briefly, the cells were subjected to oxygen-glucose deprivation for a given period and then put back to the normal culture condition (95% O_2_ and 5% CO_2_) in a normal culture medium for a specific time point (mimicking the reperfusion process). To simulate the oxygen-glucose deprivation environment, the cells were cultured with D-Hanks balanced salt solution (Biological Industries, USA) in an incubator chamber (Billups Rothenberg, Inc., Del Mar, CA) with 0.1% O_2_, 94.9% N2, and 5% CO_2_ at 37°C for 6 hours. After exposure to OGD for 6 hours, the cells were immediately put back into a normal culture medium (DMEM and 10% FBS) or conditioned medium with or without EVs, and cultured under normal conditions (37°C, 95%O_2_ and 5% CO_2_) for 0 hour, 4 hours, 24 hours, and 48 hours.

For non-OGD/R group, cells were exposed to normal control condition without OGD/R insult (37°C, 95%O_2_ and 5% CO_2_).

### 2.3. Preparation of Conditioned Medium

The conditioned media were prepared as follows: (1) N2A-CMs: when the N2A cells reached 70%-80% confluency, N2A cells were cultured with nonserum DMEM for 24 hours. Next, the conditioned medium was collected and immediately centrifuged at 1000 rpm for 10 minutes and immediately used or stored at -20°C. (2) R24H-N2A-CMs: after OGD procedure for 6 hours to cause injury in N2A cells, the D-Hank's balanced salt solution was replaced with nonserum DMEM under reperfusion condition for 24 hours, and then the conditioned medium during the reperfusion phase was collected and immediately centrifuged at 1000 rpm 10 minutes, immediately used or stored at -20°C.

For non-OGD/R group, when the UC-MSCs reached 70%-80%, they were UC-MSCs with N2A-CMs or R24H-N2A-CMs in normal control condition (without OGD/R insult, non-OGD/R condition) for 24 hours. For the OGD/R24-h group, UC-MSCs were exposed to OGD for 6 hours and then reperfused with N2A-CMs or R24H-N2A-CMs for 24 hours. The UC-MSCs were divided into five groups: (1) control group; (2) non-OGD/R (N2A-CMs) group; (3) non-OGD/R (R24H-N2A-CMs) group; (4) OGD/R (N2A-CMs) group; (5) OGD/R (R24H-N2A-CMs) group.

Subsequently, we examined cell viability, apoptosis, and oxidative stress in UC-MSCs using MTT [3-(4,5-dimethyl-2-thiazolyl)-2,5-diphenyltetrazolium bromide], Annexin-V/PI, lactate dehydrogenase (LDH) leakage, terminal deoxynucleotidyl transferase-mediated dUTP nick-end labeling (TUNEL), western blotting, and indexes of oxidative stress.

### 2.4. EVs Isolation, Identification, and Characterizations

We isolated the EVs from the N2A cells conditioned medium using ExoQuick exosome precipitation solution kit (EXOQ5A-1, Systems Biosciences, San Francisco, CA, USA) following the manufacture's protocol. Briefly, the supernatant collected from the N2A cells was centrifuged at 3000 g for 15 minutes, and the cell debris containing large vesicles were removed using a 0.2 *μ*m filter. The new supernatant was incubated with Exoquick precipitation solution at 4°C, overnight. The mixture was centrifuged at 1500 g for 30 minutes, and then at 1500 g for 5 minutes to the pellet EVs. The obtained pellet was resuspended in PBS and stored at -80°C for downstream experimentation. We used the transmission electron microscope (TEM) (Hitachi, H7650, Japan) to study the morphology of the EVs. Protein expression in EVs was analyzed using western blotting as previously described [27]. The size distribution and concentration of purified EVs were identified using Nanoparticle tracking analysis (NTA) using NanoSight (Flow Nano Analyzer, Xiamen, China).

Two kinds of EVs derived from the conditioned medium were collected from N2A cells subjected to OGD/R24H insult (R24H-N2A-CMs) or normal control condition (N2A-CMs, without OGD/R insult). Further, the concentration of the two kinds of EVs was recorded by NTA.

### 2.5. Culture of UC-MSCs with EVs Derived from N2A Cells

UC-MSCs at a confluence of 70%-80% were separately incubated in various concentrations of EVs (6 × 107 particles per ml, 1 × 107 particles per ml) in nonserum DMEM culture medium containing 10% EVs for 24 hours. For the non-OGD/R group, the UC-MSCs were divided into three groups: (1) control group; (2) EVs-1 (EVs derived from R24H-N2A-CMs) group; (3) EVs-2 (EVs derived from N2A-CMs) group.

To determine the effect of EVs on apoptosis and oxidative stress in UC-MSCs, UC-MSCs were exposed to OGD for 6 hours and then reperfused with DMEM containing two kinds of EVs for 24 hours. For the OGD/R group, the UC-MSCs were divided into four groups: (1) EVs-1 (EVs derived from R24H-N2A-CMs) group; (3) EVs-2 (EVs derived from N2A-CMs) group; (3) normal medium (normal cultured medium) group; (4) R24H-N2A-CMs group. Finally, we used MTT, LDH, Annexin-V/PI, TUNEL, western blotting, and indexes of oxidative stress to evaluate cell viability, apoptosis, and oxidative stress in UC-MSCs.

### 2.6. siRNA Transfection

Rab27a small interfering RNA and its negative control plasmid bought from GENECHEM Biotech (Shanghai, China) were transfected into the N2A cells using Lipofectamine 2000 (Invitrogen, Carlsbad, CA, United States) following the manufacturer's instructions.

### 2.7. Quantitative Real-Time (RT) PCR

We extracted total RNA from the cells using the TRIzol (Thermo-Fisher, MA, USA). A reverse transcription kit (CW Biotech, Beijing, China) was used to reverse-transcribe the RNA into cDNA. The cDNA was then used as the template for quantitative PCR using the SYBR Green PCR master mix (CW Biotech, Beijing, China). The following primer sequences were used: Rab27a: forward, 5′-TCGGGCATCCATCTGTAACGC-3′; reverse, 5′-TCCCTAAATGACCCGCCACC-3′. HIF-1*α*: forward, 5′-TCCAGCAGACCCAGTTACAGA-3′, and reverse, 5′-GCCACTGTATGCTGATGCCTT-3′. *β*-Actin: forward: 5′-ACATCCGTAAAGACCTCTATGCC-3′, and reverse, 5′-TACTCCTGCTTGCTGATCCAC-3′. The relative gene expression was calculated using the 2^-*ΔΔ*Ct^ method.

### 2.8. Apoptosis Assay

Flow cytometry was used to assess cell apoptosis with the Annexin V apoptosis assay kit, Keygen Biotech, Nanjing, China. Briefly, the cells were seeded in a 6-well plate and adhered successfully. Next, we treated the cells as per the requirements of each group. After exposure to non-OGD/R and OGD/R conditions, the Annexin V/PI solution was added into the cell solution and incubated at room temperature for 15 minutes. Finally, apoptosis was examined using a flow cytometer (FACSCalibur, Becton-Dickinson, Sunnyvale, CA).

### 2.9. Lactate Dehydrogenase (LDH) Leakage Assay

The extent of cell damage was assessed by measuring the concentration of LDH in the culture medium. Briefly, cells were exposed to non-OGD/R or OGD/R conditions, then the medium was used to assay for LDH leakage with the LDH Leakage assay kit (Beyotime, Shanghai, China) following the manufacturer's instructions. The LDH concentration was determined at 450 nm using a microplate reader (Thermo-fisher, MA, USA).

### 2.10. MTT Assay

The MTT assay used to evaluate cell viability. Briefly, the cells were seeded into 96-well plates, at a density of 1000 cells per well. The two kinds of cells (UC-MSCs and N2A cells) were cultured in the corresponding media and conditions. The MTT reagent (Beyotime, Shanghai, China) was subsequently added as per the manufacturer's descriptions [31]. Subsequently, the cells were cultured at 37°C for 4 hours. The supernatant was discarded and cells treated with dimethyl sulfoxide (DMSO) for 1 hour. Finally, the optical density (OD) of the cell media was read at 490 nm using a microplate reader (Thermo-fisher, MA, USA).

### 2.11. TUNEL Assay

TUNEL assay was performed using the TUNEL apoptosis kit (Yeason Biotech, Shanghai, China) following the manufacturer's protocol. Briefly, cells were exposed to non-OGD/R and OGD/R conditions, put on slides, and then fixed with 4% paraformaldehyde in phosphate buffer. After washing thrice with PBS, cells were permeabilized with 0.2% Triton-X 100 in methanol at 4°C for 2 minutes. The TUNEL detection reagent was then added to the cells and incubated at room temperature for 1 hour. Finally, the cell images were viewed using an immunofluorescence microscope (Olympus, Tokyo, Japan).

### 2.12. ROS, ATP, SOD, and T-AOC Assay

The level of oxidative stress was assessed by measuring the levels of ROS, ATP, SOD, and T-AOC. Cells were exposed to non-OGD/R and OGD/R condition and then assayed using the corresponding assay kits: Reactive Oxygen Species Assay Kit, ATP luminescence assay kit, Total Superoxide Dismutase Assay Kit, Total Antioxidant Capacity Assay Kit (Beyotime, Shanghai, China) following the manufacturer's instructions.

### 2.13. Western Blotting and Antibodies

We isolated total proteins from the N2A cells and UC-MSCs by treating the cells with the radio-immunoprecipitation assay (RIPA) lysis buffer (Beyotime, Shanghai, China). Next, the proteins were quantified using the BCA assay kit (Beyotime, Shanghai, China). The equal amount of proteins was separated using SDS-PAGE and transferred to the PVDF membranes. The membranes were then blocked using skimmed milk in Tris-buffered saline with Tween20 (Sinoreagent, Shanghai, China). Next, membranes were incubated with the primary antibodies overnight and then with the corresponding secondary antibodies. Finally, the blots were detected using ECL reagent (Advansta, Can, USA), analyzed, and quantified in Image J software (https://imagej.en.softonic.com). All primary and secondary antibodies used in this experiment are outlined in Supplementary Table S1.

### 2.14. Statistics Analysis

All the experimental data were expressed as the means ± SD. Student's *t*-test was used to compare the means of two groups. Multiple groups were compared using a one-way analysis of variance. The value of *P* < 0.05 was considered statistically significant. All data were analyzed using Prism 8.0 (https://www.graphpad.com) and SPSS 19.0 (https://spss.en.softonic.com).

## 3. Results

### 3.1. N2A Cells Exposed to upon OGD/R Insult Aggravated Apoptotic Death of UC-MSCs

To investigate the effect of EVs derived from injured N2A cells on the survival rate of engrafted UC-MSCs, we first tested whether OGD/R could regulate apoptosis and oxidative stress response in N2A cells and UC-MSCs in a reperfusion time-dependent manner. As shown in Supplementary Figure 1A-1F, the level of apoptosis in N2A cells and UC-MSCs was significantly higher in the OGD/R groups compared with the control group as determined by flow cytometry with Annexin-V/PI staining, LDH leakage assay, and expression of apoptosis-associated proteins detected by western blot assay (Supplementary Figure 1I-1J). Notably, cell apoptosis was the highest at the 24 hours reperfusion time-point, after which it slowly decreased in the 48 hours reperfusion time point. In the MTT assay, opposite results were obtained in the cell viability of the two kinds of cells (UC-MSCs and N2A cells), the results showed that there were obviously decreased viability of UC-MSCs and N2A cells upon OGD/R conditions relative to the control group (Supplementary Figure 1G-1H). Meanwhile, we measured the levels of oxidative stress-associated indexes ROS, ATP, SOD, and T-AOC. Results shown in the Supplementary Figure 2A and 2B reveal that the production of ROS was consistent with the findings of cell apoptosis, there were significantly upregulated in OGD/R groups compared with the control group both in UC-MSCs and N2A cells. Whereas, ATP, SOD, and T-AOC levels were significantly reduced in OGD/R groups relative to the control group. Notably, the level of ATP, SOD, and T-AOC in N2A and UC-MSCs was the lowest at 24 hours reperfusion time-point, after which it gradually increased to the 48 hours reperfusion time-point (Supplementary Figure 2C-2H), as evidenced by the firefly luciferase, WST-8 and ABTS assay, respectively. Thus, the 24 hours reperfusion duration was chosen to establish the cerebral ischemia/reperfusion *in vitro* model.

**Figure 1:**
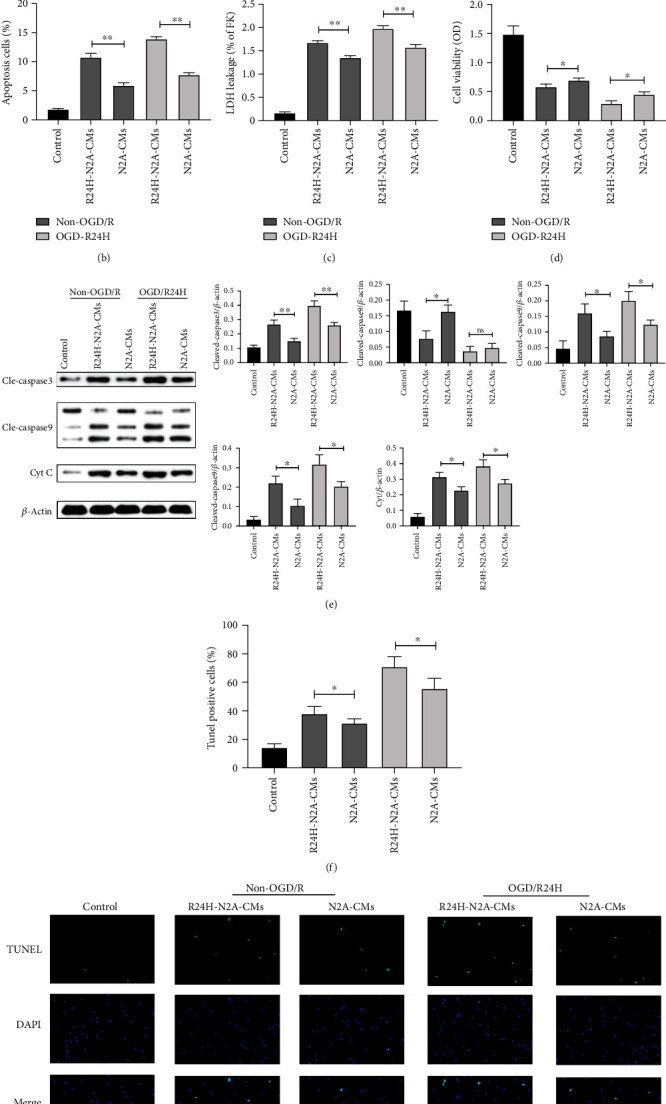
Apoptosis of UC-MSCs cultured with conditioned medium from injured N2A cells. UC-MSCs were cultured with conditioned medium from N2A cells under non-OGD/R condition or OGD/R24H insult. (a–b) Apoptosis of UC-MSCs as evaluated by flow cytometry with Annexin-V/PI staining assay. (c) Apoptosis of UC-MSCs as evaluated by LDH leakage assay. (d) Cell viability of UC-MSCs as evaluated by MTT assay. (e) Expression and quantitative data of cleaved-caspase3, cleaved-caspase9, and cytochrome C as evaluated by western blotting. (f–g) Apoptosis of UC-MSCs as evaluated by TUNEL immunofluorescence. Scale bar = 50 *μ*m. All data are presented as the mean value ± SD (*n* = 3). ∗*p* < 0.05; ∗∗*p* < 0.01, R24H-N2A-CMs group compared with N2A-CMs group.

Subsequently, UC-MSCs were cultured in the conditioned medium of the injured N2A cells that had been exposed to OGD/R24-h insult (R24H-N2A-CMs) and non-OGD/R conditions for 24 hours (N2A-CMs). Figures 1(a) and 1(b) show that the apoptosis of UC-MSCs was significantly higher in the R24H-N2A-CMs group than in the N2A-CMs group following non-OGD/R and OGD/R24-h insult. This was consistent with the result of the LDH leakage assay. The data indicates that there was an obviously increased in the R24H-N2A-CMs group compared to N2A-CMs group both upon non-OGD/R and OGD/R24-h condition (Figure 1(c)). Moreover, the MTT assay showed that cell viability was significantly lower in the R24H-N2A-CMs group both in the non-OGD/R or OGD/R24-h insult conditions than in N2A-CMs (Figure 1(d)). The expression level of proteins associated with apoptosis (cleaved-caspase3, cleaved-caspase9, and cytochrome C) was significantly higher in the R24H-N2A-CMs group than in the N2A-CMs group in non-OGD/R and OGD/R24-h conditions (Figure 1(e)). The TUNEL assay revealed that the percentages of TUNEL positive UC-MSCs were significantly higher in the R24H-N2A-CMs group than in the N2A-CMs group in non-OGD/R and OGD/R24-h conditions (Figures 1(f) and 1(g)). Altogether, these findings revealed that the conditioned medium from injured N2A cells aggravated the apoptosis of UC-MSCs in the context of OGD/R insult.

### 3.2. Culture of UC-MSCs with Conditioned Medium from Injured N2A Cells Exacerbated Oxidative Stress Response

**Figure 2:**
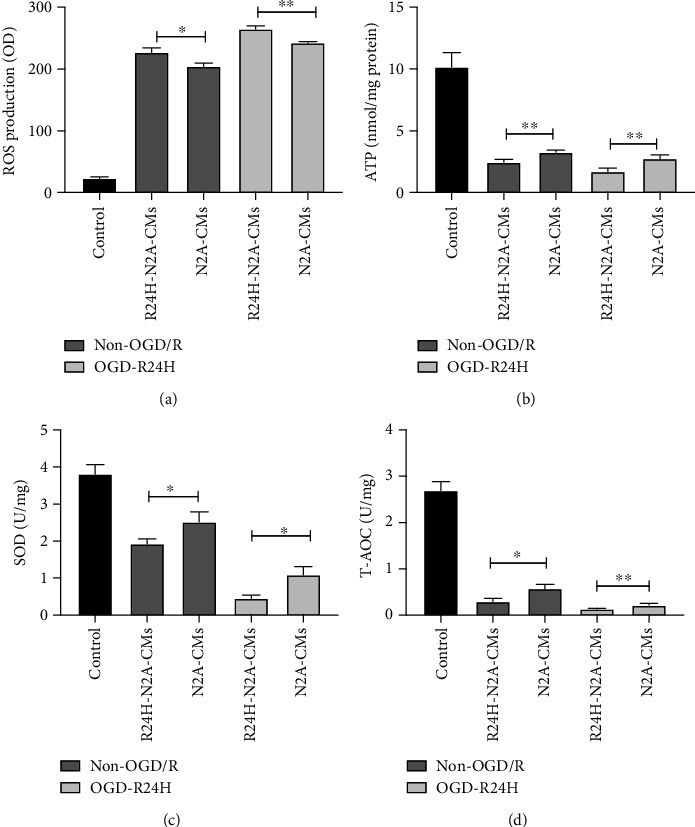
Oxidative stress response of UC-MSCs cultured with conditioned medium from injured N2A cells. UC-MSCs were cultured with conditioned medium from N2A cells under non-OGD/R condition or OGD/R24H injury. (a) Levels of ROS as determined by dihydroethidium-DCFH-DA assay. (b) Levels of ATP as determined by firefly luciferase assay. (c) Levels of SOD as determined by WST-8 assay. (d) Levels of T-AOC as determined by ABTS assay. All data are presented as the mean value ± SD (*n* = 3). ∗*p* < 0.05; ∗∗*p* < 0.01, R24H-N2A-CMs group compared with N2A-CMs group.

Results shown in Figure 2(a) revealed that the ROS level was significantly higher in R24H-N2A-CMs group than in N2A-CMs group both under non-OGD/R or OGD/R24-h group, as determined by dihydroethidium-DCFH-DA assay. By contrast, the levels of ATP and SOD were significantly lower in R24H-N2A-CMs group compared to N2A-CMs group both under OGD/R24-h and non-OGD/R conditions (Figures 2(b) and 2(c)), as demonstrated by firefly luciferase assay and WST-8 assay. Moreover, we found that T-AOC was markedly lower in R24H-N2A-CMs group than in N2A-CMs group both under non-OGD/R or OGD/R24H conditions (Figure 2(d)). In summary, the culture of UC-MSCs in conditioned medium from injured N2A cells promoted oxidative stress level of engrafted UC-MSCs under OGD/R condition. These results indicated that the low survival of transplanted UC-MSCs may be induced by substances in the conditioned medium of the injured N2A cells through paracrine function.

### 3.3. Identification and Characterization of EVs Derived from N2A Cells

**Figure 3:**
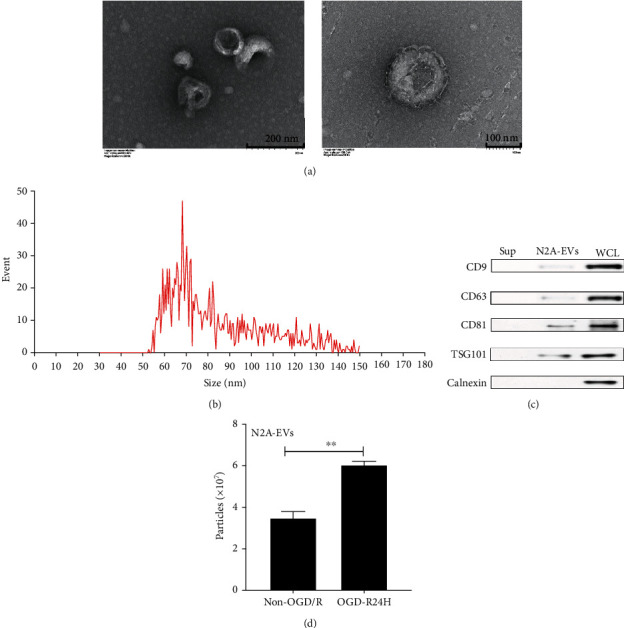
Identification and characterization of EVs derived from N2A cells. (a) Representative image of EVs by transmission electron microscopy (TEM) scale bar, 200 nm and 100 nm. (b) The size distribution of EVs by nanosight tracking analysis (NTA). (c) Western blotting analysis of EVs markers in supernatant (Sup), EVs, and whole cellular lysis (WCL). (d) The concentration of EVs from N2A cells conditioned medium upon non-OGD/R condition and OGD/R24H insult as evaluated by NTA. All data are presented as the mean value ± SD (*n* = 3). ∗*p* < 0.05; ∗∗*p* < 0.01. non-OGD/R group compared with OGD/R24H group.

Particles extracted from the conditioned medium of N2A cells were characterized with transmission electron microscopy (TEM), NTA, and western blotting assays. Morphologically, the particles appeared round or cup shaped, with a diameter ranging from 30 to 150 nm (Figures 3(a) and 3(b)), as detected by TEM and NTA. The EVs markers, CD63, CD9, CD81, and TSG101 were positively presented on the particles as revealed by western blotting (Figure 3(c)). In contrast, the expression of calnexin was not detected in the particles (Figure 3(c)). In addition, the number of particles from N2A cells was significantly higher in OGD/R24-h group than in non-OGD/R group (Figure 3(d)). These results confirmed that the particles from N2A cells were EVs, and that OGD/R induced secretion of EVs from N2A cells.

### 3.4. EVs Derived from Injured N2A Cells Enhanced Apoptotic Death of UC-MSCs

**Figure 4:**
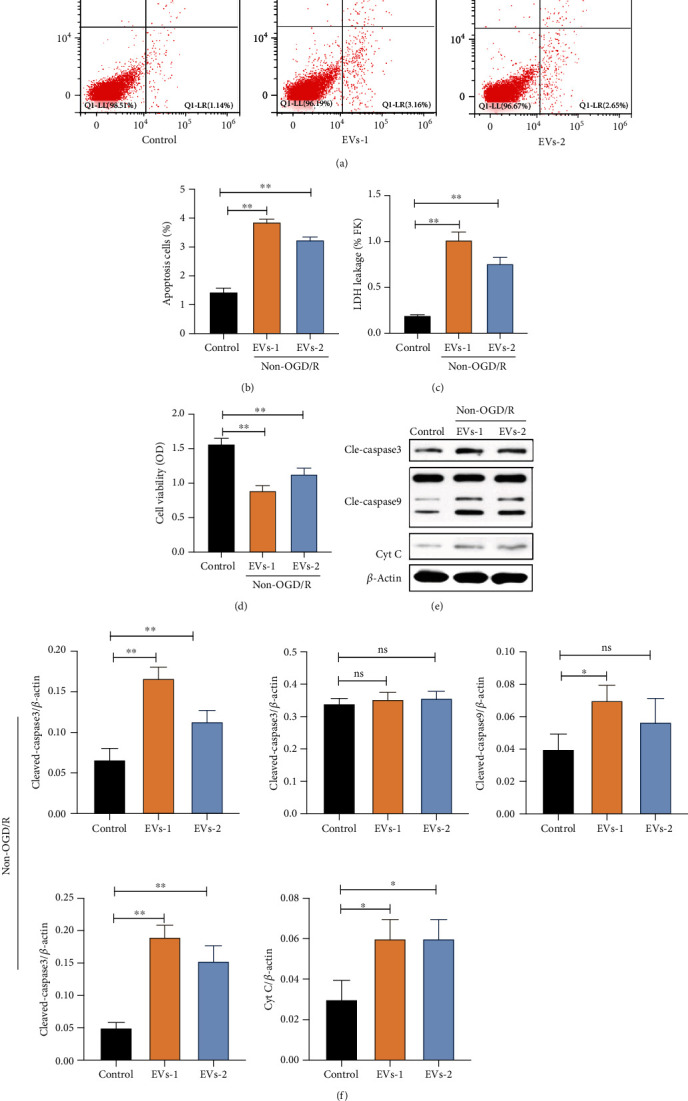
Effects of EVs derived from N2A cells on apoptosis of UC-MSCs upon non-OGD/R condition. UC-MSCs were cultured with two various concentration EVs under non-OGD/R condition for 24 hours. (a–b) Apoptosis of UC-MSCs as evaluated by flow cytometry with Annexin-V/PI staining assay. (c) Apoptosis of UC-MSCs as evaluated by LDH leakage assay. (d) Cell viability of UC-MSCs as evaluated by MTT assay. (e–f) Expression and quantitative data of cleaved-caspase3, cleaved-caspase9, and cytochrome C as evaluated by western blotting. EVs-1: represented as higher concentration EVs recorded by NTA assay. EVs-2: represented as lower concentration EVs recorded by NTA assay. All data are presented as the mean value ± SD (*n* = 3). ∗*p* < 0.05; ∗∗*p* < 0.01, compared with the control group.

Next, we explored the impact of EVs derived from the injured N2A cells on the apoptosis of UC-MSCs. UC-MSCs were preincubated with two EVs concentrations (6 × 107 particles per ml, 1 × 107 particles per ml) for 24 hours to allow EVs uptake. Under non-OGD/R condition, apoptosis and LHD leakage from UC-MSCs were significantly higher in both concentrations of EVs compared with the control group (Figures 4(a)–4(c)). In contrast, the viability of UC-MSCs was significantly decreased in both concentrations of EVs compared with the control group (Figure 4(d)). Moreover, the expression of cleaved-caspase3, cleaved-caspase9, and cytochrome C was markedly higher in UC-MSCs cultured with the two EVs concentrations relative to the control group under non-OGD/R condition (Figures 4(e) and 4(f)). Notably, apoptosis was obviously severe in the high EVs concentration group than in the low EVs concentration group (Figure 4).

**Figure 5:**
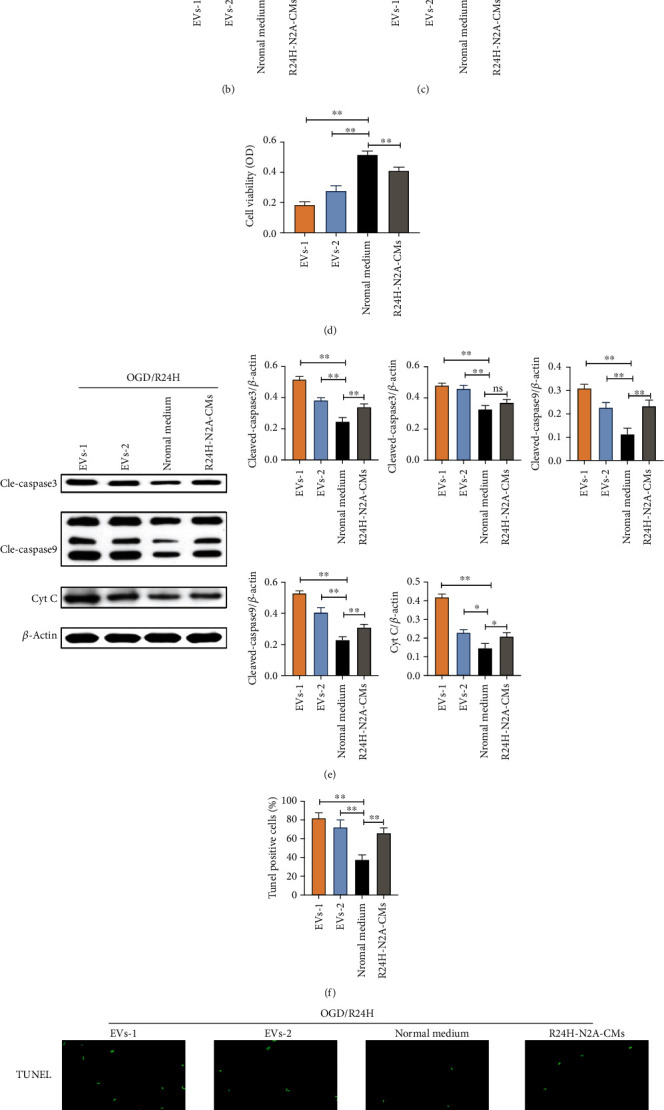
Effects of EVs derived from N2A cells on apoptosis of UC-MSCs upon OGD/R24H insult. UC-MSCs were precultured with two various concentration EVs, normal cultured medium, and R24H-N2A-CMs for 24 hours, respectively. Following treatment, UC-MSCs were subjected to OGD/R24H insult. (a–b) Apoptosis of UC-MSCs as evaluated by flow cytometry with Annexin-V/PI staining assay. (c) Apoptosis of UC-MSCs as evaluated by LDH leakage assay. (d) Cell viability of UC-MSCs as evaluated by MTT assay. (e) Expression and quantitative data of cleaved-caspase3, cleaved-caspase9, and cytochrome C as evaluated by western blotting. (f–g) Apoptosis of UC-MSCs as evaluated by TUNEL immunofluorescence. Scale bar = 50 *μ*m. EVs-1: represented as higher concentration EVs recorded by NTA assay. EVs-2: represented as lower concentration EVs recorded by NTA assay. All data are presented as the mean value ± SD (*n* = 3). ∗*p* < 0.05; ∗∗*p* < 0.01, compared with normal culture medium group.

Further experiments were conducted to evaluate the effect of EVs derived from N2A cells on UC-MSCs apoptosis and oxidative stress response. Flow cytometry with Annexin-V/PI staining assay results showed that the apoptosis was lowest in UC-MSCs cells cultured with normal cultured medium compared to other groups (Figures 5(a) and 5(b)). In addition, apoptosis level and LHD leakage from UC-MSCs were significantly higher in the two concentrations of EVs under OGD/R24-h conditions compared to the control group (Figure 5(c)). We also observed that OGD/R24-h injury resulted in significantly lower viability of UC-MSCs cells in both EVs concentration compared with UC-MSCs cultured in normal cultured medium (Figure 5(d)). Moreover, the protein levels of cleaved-caspase3, cleaved-caspase9, and cytochrome C were markedly higher in UC-MSCs cultured with EVs under OGD/R24-h injury than those cultured in normal cultured medium (Figure 5(e)). UC-MSCs treated with a higher concentration of EVs had obviously larger number of TUNEL positive cells than those treated with a lower concentration of EVs under OGD/R24-h condition (Figures 5(f) and 5(g)). These results suggested that EVs derived from injured N2A cells aggravated apoptosis of UC-MSCs.

### 3.5. EVs Derived from Injured N2A Cells Promoted Oxidative Stress Level of UC-MSCs

**Figure 6:**
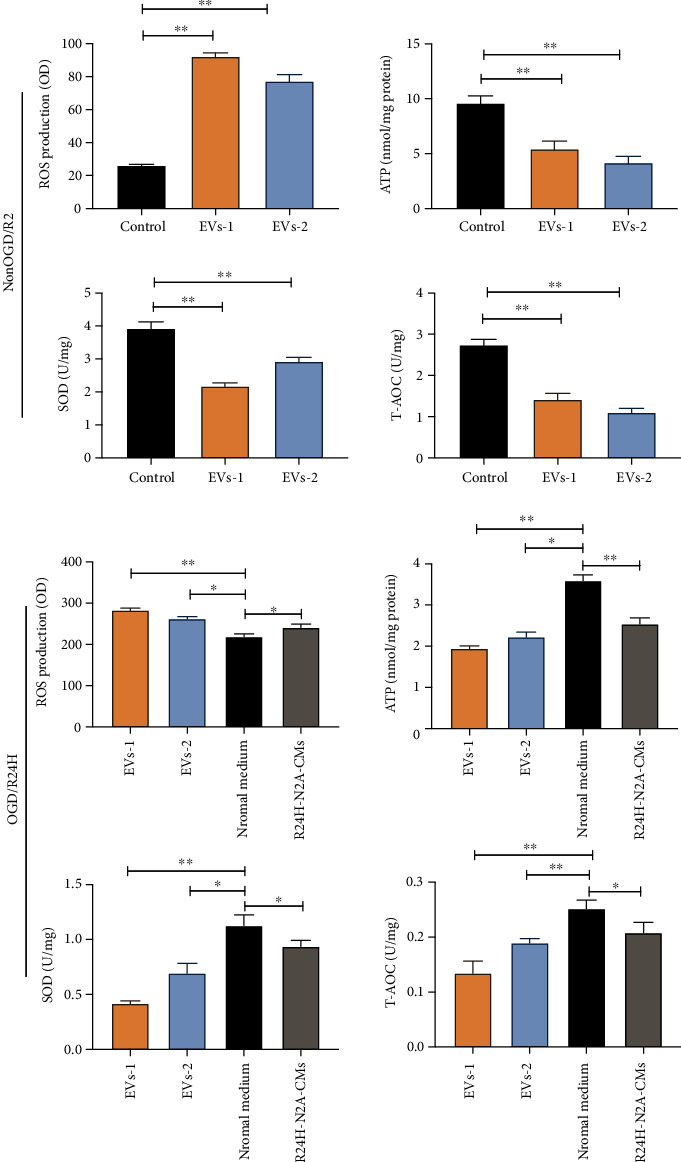
Effect of EVs derived from N2A cells on oxidative stress level of UC-MSCs. UC-MSCs were cultured with two various concentration EVs under non-OGD/R condition and OGD/R24H insult, respectively. (a) Levels of ROS as determined by dihydroethidium-DCFH-DA (DHE) under non-OGD/R condition. (b) Levels of as determined by firefly luciferase assay under non-OGD/R condition. (c) Levels of SOD as determined by WST-8 assay under non-OGD/R condition. (d) Levels of T-AOC as determined by ABTS assay under non-OGD/R condition. (e) Levels of ROS as determined by dihydroethidium-DCFH-DA assay upon OGD/R24H insult. (f) Levels of ATP as detected by firefly luciferase assay upon OGD/R24H insult. (g) Levels of SOD as determined by WST-8 assay upon OGD/R24H insult. (h) Levels of T-AOC as determined by ABTS assay upon OGD/R24H insult. EVs-1: represented as higher concentration EVs recorded by NTA assay. EVs-2: represented as lower concentration EVs recorded by NTA assay. All data are presented as the mean value ± SD (*n* = 3). ∗*p* < 0.05; ∗∗*p* < 0.01, compared with control group or normal culture medium group.

Next, we explored the impact of EVs on the oxidative stress level of UC-MSCs. Under non-OGD/R condition, results showed that ROS level was significantly higher in UC-MSCs treated with both concentrations of EVs compared with UC-MSCs from the control group (Figure 6(a)), while ATP, SOD, and T-AOC levels were markedly lower in UC-MSCs treated with both concentrations of EVs relative to the control group (Figures 6(b)–6(d)). Next, we exposed UC-MSCs pretreated with various concentration EVs to OGD/R under conditions. Results showed that treatment with EVs led to significantly higher levels of ROS in UC-MSCs compared to UC-MSCs cultured in normal culture medium under OGD/R24-h injury. In contrast, the level of ATP, SOD, and T-AOC in UC-MSCs treated with EVs groups were markedly lower than in UC-MSCs cultured with normal cultured medium under OGD/R24-h injury (Figures 6(f)–6(h)). Moreover, UC-MSCs treated with higher EVs concentration showed obviously severe oxidative stress response than those treated with lower EVs concentration under non-OGD/R and OGD/R conditions. Collectively, these data demonstrated that EVs from injured N2A cells enhanced the severity of oxidative stress in UC-MSCs under OGD/R injury. These indicated that the EVs affected the survival of engrafted UC-MSCs.

### 3.6. Suppression of EVs Release from N2A Cell Alleviated Apoptosis in UC-MSCs

**Figure 7:**
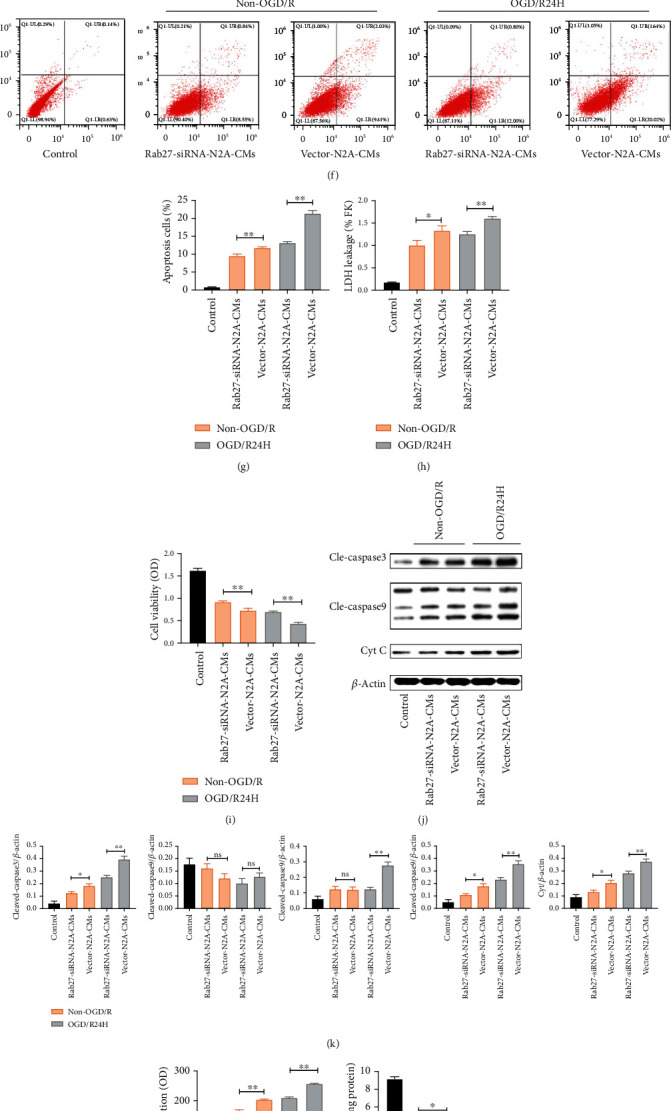
Impact of silencing Rab27a on the release of EVs from N2A cells and injury to UC-MSCs. N2A cells were transfected with Rab27a siRNA. These cells were verified with western blotting, mRNA and EVs markers. (a–c) Silencing of Rab27a on N2A cells as evaluated by western blotting and mRNA. (d) Western blotting analysis of EVs markers in EVs and whole cellular lysis (WCL). (e) The concentration of EVs as evaluated by NTA. Next, UC-MSCs were cultured with medium from Rab27a-siRNA-CMs group and Vector-N2A-CMs group under non-OGD/R condition and OGD/R24H insult. (f–g) Apoptosis of UC-MSCs in Rab27a-siRNA-CMs group and Vector-N2A-CMs group mediums under non-OGD/R condition and OGD/R24H insult were detected by flow cytometry assay. (h) Apoptosis of UC-MSCs as detected by LDH leakage assay. (i) Cell viability of UC-MSCs as determined by MTT assay. (j–k) Expression and quantitative data of cleaved-caspase3, cleaved-caspase9, and cytochrome C were determined by western blotting. (l) Levels of ROS as determined by dihydroethidium-DCFH-DA assay. (m) Levels of ATP as determined by firefly luciferase assay. (n) Levels of SOD as determined by WST-8 assay. (O) Levels of T-AOC as detected by ABTS assay. All data are presented as the mean value ± SD (*n* = 3). ∗*p* < 0.05; ∗∗*p* < 0.01, control group compared with Rab27s siRNA group. Rab27a-siRNA-N2A-CMs group compared with Vector-N2A-CMs group.

Studies have been reported that Rab27a regulated EVs secretion [32]. To confirm the function and mechanism by which EVs promoted injury to the transplanted UC-MSCs, we suppressed EVs release by silencing Rab27a in N2A cells. Results showed that Rab27a knock-down in N2A cells significantly decreased the protein and mRNA levels compared to the control group (Figures 7(a)–7(c)). Notably, Rab27a-siRNA markedly decreased expression of EVs markers CD9, CD63, TSG101, and CD81 relative to the control group (Figure 7(d)). However, the expression of calnexin was not markedly altered following in the whole cellular lysis control group and Rab27a silencing group (Figure 7(d)). In contrast, the expression of calnexin was not detected in the EVs control group and Rab27a-siRNA group (Figure 7(d)). In addition, Rab27a-knock-down N2A cells significantly decreased the number of EVs released as revealed by NTA (Figure 7(e)). These datasets prove that Rab27a-knock-down obviously inhibited the release of EVs from N2A cells.

Next, we determined the effect of Rab27a-siRNA on apoptosis of transplanted UC-MSCs under non-OGD/R and OGD/R24-h conditions. Indeed, suppression of EVs secretion obviously inhibited apoptosis and LDH leakage in Rab27a-siRNA treated group compared to the vector group, both in non-OGD/R and OGD/R24-h condition (Figures 7(f)–7(h)). In addition, silencing of Rab27a significantly enhanced the viability of UC-MSCs both in non-OGD/R and OGD/R24-h conditions compared to the vector group (Figure 7(i)). As expected, the protein levels of cleaved-caspase3, cleaved-caspase9, and cytochrome C were also significantly decreased in UC-MSCs after Rab27a depletion under non-OGD/R and OGD/R conditions relative to the vector group (Figure 7(j)). Taken together, these results strongly show that inhibition of EVs released from N2A cells prevents apoptosis of UC-MSCs following cerebral ischemia/reperfusion injury.

### 3.7. Inhibition of EVs Secretion from N2A Cells Reduced Oxidative Stress in UC-MSCs

We further explored whether reducing EVs secretion from N2A cells could protect against oxidative stress in engrafting UC-MSCs upon non-OGD/R injury. Indeed, silencing of Rab27a in N2A cells resulted in a dramatic downregulation of ROS both under non-OGD/R and OGD/R24-h conditions compared to the vector group (Figure 7(k)). Further, Rab27a silencing strikingly elevated the production of ATP and SOD in UC-MSCs both under non-OGD/R and OGD/R24-h conditions compared to the vector group, accompanied by significantly increased T-AOC production unlike in the vector group (Figures 7(l)–7(o)). These data suggest that reducing the release of EVs from N2A cells could suppress oxidative stress and improve the survival of transplanted UC-MSCs in the context of OGD/R injury.

### 3.8. Hypoxia Preconditioning Enhanced the Survival of UC-MSCs upon OGD/R Insult

**Figure 8:**
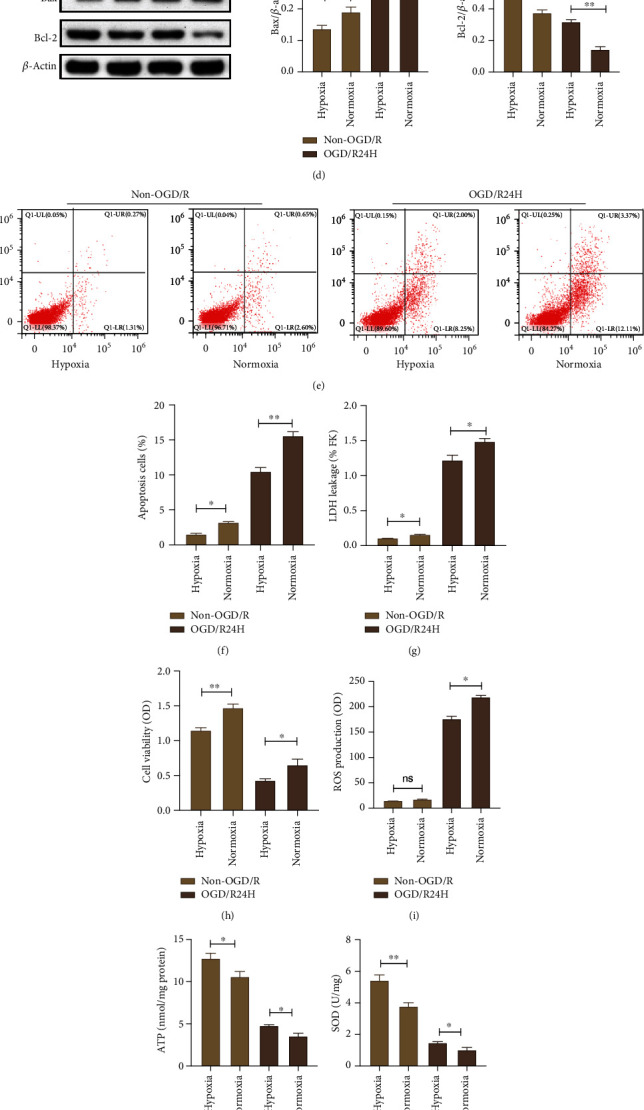
Hypoxia preconditioned enhances the survival of UC-MSCs upon OGD/R insult. UC-MSCs were cultured under hypoxia or normoxia preconditioning before non-OGD/R and OGD/R insult. (a–c) The expression of HIF-1*α* in normoxia and hypoxia preconditioned UC-MSCs group as evaluated western blotting and mRNA. (d) Expression and quantitative data of bax and bcl-2 were determined by western blotting. (e–f) Apoptosis of UC-MSCs as assessed by flow cytometry assay. (g) Apoptosis of UC-MSCs as determined by LDH leakage assay. (h) Cell viability of UC-MSCs as examined by MTT assay. (i) Levels of ROS as determined by dihydroethidium-DCFH-DA assay. (j) Levels of ATP as determined by firefly luciferase assay. (k) Levels of SOD as examined by WST-8 assay. (l) Levels of T-AOC as measured by ABTS assay. All data are presented as the mean value ± SD (*n* = 3). ∗*p* < 0.05; ∗∗*p* < 0.01, hypoxia group compared with normoxia group.

Hypoxia preconditioning MSCs has been suggested to enhance the efficacy and survival of engrafted MSCs in several diseases including cerebral ischemic stroke. These beneficial effects are mediated by hypoxia inducible factor (HIF-1*α*) [33]. Therefore, we investigated whether hypoxia preconditioning could promote the survival of UC-MSCs. The expression of HIF-1*α* was quantified by qPCR and western blot analysis. We found that protein and mRNA expression of HIF-1*α* was significantly higher in hypoxia preconditioned UC-MSCs group compared to UC-MSCs subjected to normoxia preconditioned group (Figures 8(a)–8(c)). Then, we examined the effect of hypoxia preconditioning on apoptosis and oxidative stress level of UC-MSCs. The protein expression of bax was significantly lower while that of bcl-2 was significantly higher in hypoxia preconditioned UC-MSCs unlike in normoxia preconditioned UC-MSCs group both under non-OGD/R and OGD/R24-h conditions (Figure 8(d)). Additionally, the cell apoptosis and LHD leakage were markedly lower in hypoxia precondition group under non-OGD/R and OGD/R24-h conditions than in normoxia preconditioned group (Figures 8(e)–8(g)), whereas the cell viability showed obviously opposite results, the findings showed that there was a significantly higher viability of UC-MSCs in hypoxia pretreatment group compared to normoxia pretreatment group either upon non-OGD/R or OGD/R24-h conditions (Figure 8(h)).

Furthermore, results shown in Figure 8(i) revealed that the level of ROS was markedly lower in hypoxia preconditioned group under non-OGD/R and OGD/R24-h conditions than in normoxia group. In contrast, ATP, SOD, and T-AOC were significantly higher in hypoxia preconditioned group under non-OGD/R and OGD/R24-h conditions relative to normoxia preconditioned group (Figures 8(j)–8(l)). Collectively, these results suggest that hypoxia preconditioning promoted the survival of transplanted UC-MSCs by reducing apoptosis and oxidative stress response of UC-MSCs under cerebral ischemia/reperfusion injury.

### 3.9. Hypoxia Preconditioning Protected UC-MSCs against the Paracrine Action of the Injured N2A Cells

**Figure 9:**
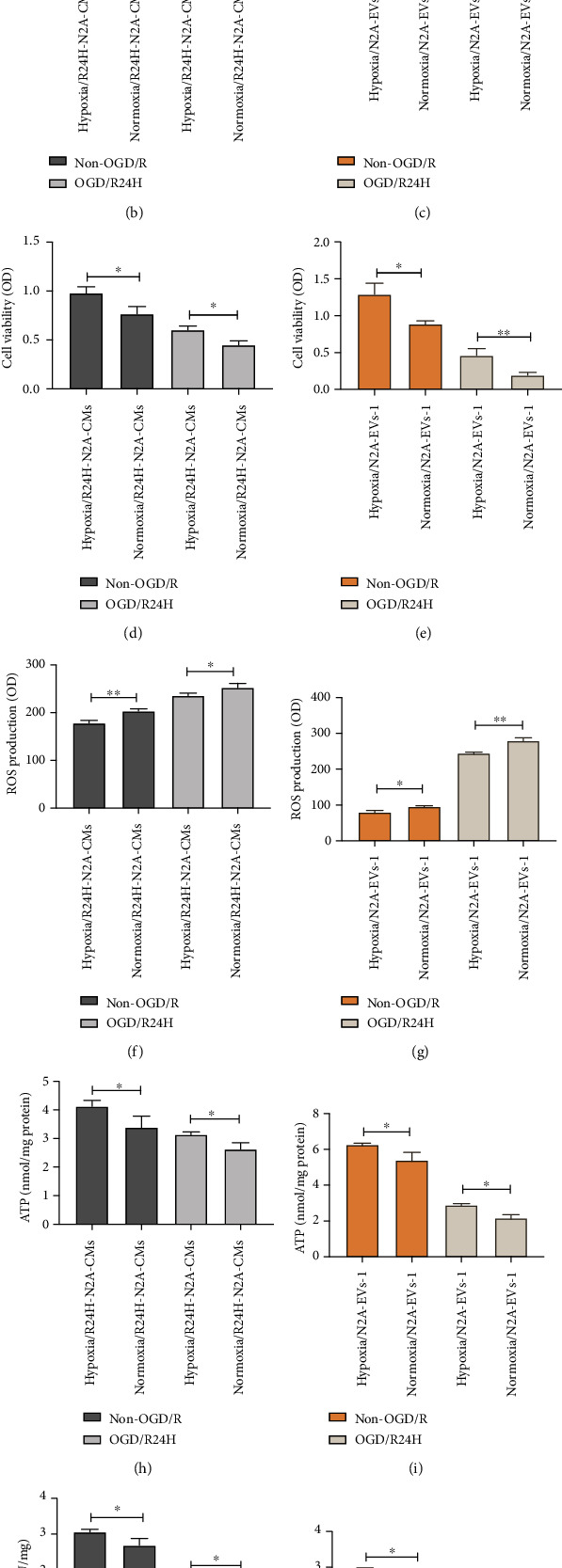
Effect of hypoxia preconditioned UC-MSCs on the paracrine action of EVs from injured N2A cells. Hypoxia or normoxia preconditioned UC-MSCs were cultured with conditioned medium (R24H-N2A-CMs) and high concentration EVs (EVs-1) from injured N2A cells. (a) Expression and quantitative data of bax, bcl-2, cleaved-caspase3, cleaved-caspase9, and cytochrome C in each experiment group were determined by western blotting. (b–c) Apoptosis of UC-MSCs as quantified by LDH leakage assay. (d–e) Cell viability of UC-MSCs as determined by MTT assay. (f–g) Levels of ROS as evaluated by dihydroethidium-DCFH-DA assay. (h–i) Levels of ATP as detected by firefly luciferase assay. (j–k) Levels of SOD as assessed by WST-8 assay. (l–m) Levels of T-AOC as detected by ABTS assay. All data are presented as the mean value ± SD (*n* = 3). ∗*p* < 0.05; ∗∗*p* < 0.01, hypoxia group compared with normoxia group.

We subsequently investigated the mechanism by which hypoxia preconditioned UC-MSCs affected the paracrine function of injured N2A cells under OGD/R condition. Results shown in Figures 9(a)–9(c) showed that the expression of apoptosis-associated proteins (bax, cleaved-caspase3, and cleaved caspase 9) was significantly lower, and the expression of bcl-2 was significantly lower in hypoxia preconditioned group in cells cultured with R24H-N2A-CMs or high concentration EVs (EVs-1) under non-OGD/R and OGD/R24-h conditions than in normoxia preconditioned group (Figure 9(a)). These results were consisted with the findings of LDH leakage assay. Figures 9(b) and 9(c) showed that there was an obviously reduced in LDH leakage in hypoxia preconditioned group in OM-MSCs treatment with R24H-N2A-CMs or high concentration EVs (EVs-1) under non-OGD/R and OGD/R24-h conditions compared to normoxia preconditioned group. Moreover, the viability of UC-MSCs was significantly higher in hypoxia preconditioned group in UC-MSCs cocultured with R24H-N2A-CMs and high concentration EVs (EVs-1) under non-OGD/R and OGD/R24-h conditions relative to normoxia preconditioned group (Figures 9(d) and 9(e)).

In addition, the level of ROS was significantly lower in hypoxia precondition group in UC-MSCs cocultured with R24H-N2A-CMs and high concentration EVs (EVs-1) under non-OGD/R and OGD/R24-h injury than in normoxia preconditioned UC-MSCs group (Figures 9(f) and 9(g)). In contrast, levels of ATP, SOD, and T-AOC were markedly elevated in hypoxia precondition group relative to normoxia preconditioned group in the group cocultured with R24H-N2A-CMs and high concentration EVs under non-OGD/R and OGD/R24-h injury (Figures 9(h)–9(m)). Taken together, these datasets suggest that hypoxia preconditioning enhanced the survival of UC-MSCs to protect against the paracrine effect of N2A cells injured by cerebral ischemia/reperfusion *in vitro*.

## 4. Discussion

Cerebral ischemic stroke continues to be an intractable challenge in clinical practice. Recent studies have shown that MSCs-based therapies effectively control cerebral ischemia/reperfusion insult [34]. However, when transplanted, MSCs face harsh microenvironment created by ischemia/reperfusion insult, such as high oxidative stress, nutrition deficiency among other factors [35]. Thus, the survival rate of transplanted MSCs is relatively low which subtracts the benefits of MSCs therapy in controlling cerebral ischemic stroke.

In this study, we first explored whether OGD/R induces apoptosis and oxidative stress in N2A cells and transplanted UC-MSCs. Results showed that OGD/R insult induced significant apoptotic cell death and elevated oxidative stress levels in N2A cells and UC-MSCs during reperfusion in a time-dependent manner (Supplementary Figures 1 and 2). These effects were absent in the control group cells. Consistent with previous findings in primary neurons [36], OGD/R induced apoptosis and oxidative stress in N2A cells. Other studies have shown that transplanted neural stem cells undergo apoptosis or cell death under cerebral ischemia/reperfusion conditions before they differentiate and migrate, rendering them unable to enhance neuro-regeneration. This is also consistent with our findings in UC-MSCs. Given the harsh microenvironment caused by ischemia/reperfusion insult, it is likely that the transplanted UC-MSC cannot survive [35]. This calls for the development of strategies to improve the survival of UC-MSCs under cerebral ischemia/reperfusion environment.

Some scholars hold the view that the neuroprotective role of MSCs is mediated by paracrine mechanisms rather than their capacity to replace damaged cells [37, 38]. Other studies indicate that EVs released from MSCs function in a paracrine manner to mediate the effects of MSCs [38]. EVs are released by various types of cells. Several studies have reported that EVs are cargo-carriers participating in cell to cell communication. They also play a critical role in neurogenesis and angiogenesis in several neurological diseases [39, 40]. MSCs-derived EVs have been suggested to have therapeutic effects in cerebral ischemia stroke owing to their ability to restore and regenerate injured neurons. This is because they can cross the blood-brain barrier [36, 41, 42]. Several types of cells in the nervous system, including neurons, microglial and glia cells secrete EVs. However, evidence that EVs derived from injured neural cells influence the transplanted UC-MSCs following cerebral ischemia/reperfusion is scarce. Therefore, we aimed to investigate whether treatment with EVs secreted from injured neuronal cells could improve MSCs treatment after cerebral ischemic stroke.

Thus, we cultured UC-MSCs in a conditioned medium from injured N2A cells exposed to OGD/R insult. Results showed that the rate of apoptosis, LDH leakage, and the expression of apoptosis-associated proteins were significantly higher, while cell viability was lower in R24H-N2A-CMs group both under OGD/R and non-OGD/R condition compared to N2A-CMs group (Figure 1). Meanwhile, the level of ROS was markedly higher while levels of ATP, SOD, and T-AOC were obviously lower in R24H-N2A-CMs group relative to N2A-CMs group both under OGD/R and non-OGD/R condition (Figure 2). Further analysis revealed that the level of injury in R24H-N2A-CMs group was more severe under OGD/R condition than in R24H-N2A-CMs group under non-OGD/R condition. These findings indicated that the conditioned medium from the injured N2A cells aggravated OGD/R-induced apoptotic death of UC-MSCs. Compared to the neuroprotective role of EVs released by neuronal, these results suggested that EVs isolated from the conditioned medium may also secrete harmful proteins or nuclei acids under harsh OGD/R environment.

To test this possibility, we pretreated UC-MSCs with EVs isolated from the conditioned medium of injured N2A cells. As expected, our experiments showed that under either non-OGD/R or OGD/R condition, pretreatment of UC-MSCs with EVs obviously exacerbated apoptotic cell death, oxidative stress, and the decline in cell viability of UC-MSCs compared to cells treated with normal culture medium (Figures 4–6). Moreover, UC-MSCs cultured with high concentration EVs showed worse outcomes than those incubated with low concentration of EVs (Figures 4–6). These results indicate that EVs derived from the injured N2A cells may have adverse effects compared to normal N2A cells which protect the nervous system. Our results are in agreement with previous reports in neurodegeneration disorders suggested that EVs also promote the secretion of toxic oligomers from protein aggregates [43]. In summary, these results demonstrate that EVs from N2A cells aggravate OGD/R-induced injury and thus the survival of engrafted UC-MSCs.

We further investigated the mechanism by which the EVs aggravated the injury caused by OGD/R. In previous reports, Rab27a was found to regulate the secretion of EVs [32]. Thus, silencing of Rab27a may suppress the release of N2A-EVs. Consistent with previous research [21], our results indicated that the treatment of N2A cells with Rab27a-siRNA decreased the protein level of classical EVs markers CD9, CD63, Tsg101, and CD81 and the number of EVs secreted compared to the vector group (Figure 7). Next, UC-MSCs were cultured with conditioned medium from Rab27a-siRNA N2A cells. Results showed that UC-MSCs exposed to Rab27a-siRNA N2A cells had significantly lower apoptosis or oxidative stress levels compared with those exposed to N2A cells transfected with vector both under non-OGD/R and OGD/R conditions (Figure 7). These findings are consistent with a previous study which found that EVs secreted by neurons from traumatic spinal cord injury modulated neuroinflammation thereby promoting microgliosis and astrogliosis [18]. Hara et al. suggested that the release of EVs may be a mechanism used to maintain homeostasis of the secreting cells. The EVs contain DNA fragments and various deleterious cellular constituents [21]. Further, it was found that suppression of EVs secretion from normal or senescent cells resulted in DNA damage due to excessive production of ROS [21], which is suggested to be an adaptive response to stress [44]. Previous findings and the current results reveal that EVs derived from injured N2A cells aggravate apoptosis and oxidative stress in transplanted UC-MSCs. We postulate that the harmful constituents released from injured N2A cells may be a self-regulatory and protective mechanism in response to the harsh microenvironment. Overall, our experiments strongly show that EVs from injured N2A cells improves the survival rate of engrafted UC-MSCs. However, the detailed signaling pathways and underlying mechanisms require further exploration.

Accumulating evidence indicates that hypoxia preconditioning promotes the therapeutic effects and survival of transplanted MSCs [27, 45]. However, whether hypoxia preconditioning could improve survival of transplanted UC-MSCs under cerebral ischemia/reperfusion context deserves further investigation. The protein and mRNA expression of HIF-1*α* were significantly higher in UC-MSCs exposed to hypoxia preconditioned conditions than in UC-MSCs exposed to normoxia preconditioned conditions (Figure 8). This is consistent with a previous study on bone mesenchymal stem cells [33, 45]. Figure 8 showed that the apoptotic death of UC-MSCs and oxidative stress level were significantly lower in hypoxia preconditioned group than in normoxia preconditioned group both under non-OGD/R and OGD/R24-h conditions. Consistent with previous reports [23], we found that HIF-1*α* maintains differentiation and proliferation of cells. Given the protection role of hypoxia preconditioning, we explored whether hypoxia preconditioning could increase the tolerance of UC-MCs against the paracrine action of substances released from injured N2A cells. Thus, UC-MSCs were exposed to hypoxia preconditioning for 24 hours and then cultured with a conditioned medium from injured N2A cells. Results demonstrated that apoptosis cell death and oxidative stress level were significantly lower in UC-MSCs subjected to hypoxia preconditioning relative to those exposed to normoxia conditions both in the conditioned medium or high concentration EVs (Figure 9). These findings are in agreement with reports by Guo et al. who found that hypoxia preconditioning augmented the treatment efficacy of MSCs in cerebral ischemia/reperfusion injury [46]. Other studies have suggested that hypoxia preconditioning of MSCs may promote their paracrine functions through the release of several growth factors and anti-inflammatory factors in EVs [27, 47]. Thus, hypoxia preconditioned UC-MSCs have enhanced capacity to survive under OGD/R conditions in neuronal cells. Taken together, we have shown that hypoxia preconditioning enhances the survival of transplanted UC-MSCs. Moreover, we show that the paracrine effects of nerve cells affect the survival-promoting effects of hypoxia preconditioning on transplanted UC-MSCs.

## 5. Conclusions

In conclusion, this study shows that EVs from injured nerve cells may aggravate OGD/R-induced insult on transplanted UC-MSCs. This explains the low survival rate of engrafted UC-MSCs. In addition, our data indicates that hypoxia preconditioning may promote the survival of engrafted UC-MSCs by suppressing the paracrine mechanism of injured neuronal cells. Although this study was based on cell experiments, our findings provide a promising approach for clinical treatment of cerebral ischemic stroke.

## Data Availability

The authors declare that all data supporting the findings of this study are available within the paper and its supplementary information files.

## Supplementary Materials

Supplementary Materials Supplementary Table 1: primary and secondary antibodies. Supplementary Figure 1: OGD/R induced apoptosis of N2A cells and UC-MSCs during reperfusion in a time-dependent manner. Supplementary Figure 2: OGD/R induced oxidative stress in N2A cells and UC-MSCs during reperfusion in a time-dependent manner.
