# Supplementary material for: Effects of the Insulted Neuronal Cells-Derived Extracellular Vesicles on the Survival of Umbilical Cord-Derived Mesenchymal Stem Cells following Cerebral Ischemia/Reperfusion Injury
Source: Oxid Med Cell Longev. 2020 Jul 16;2020:9768713. doi: 10.1155/2020/9768713 (PMC7382764; doi:10.1155/2020/9768713)
Supplement: Supplementary Materials — Supplementary Table 1: primary and secondary antibodies. Supplementary Figure 1: OGD/R induced apoptosis of N2A cells and UC-MSCs during reperfusion in a time-dependent manner. Supplementary Figure 2: OGD/R induced oxidative stress in N2A cells and UC-MSCs during reperfusion in a time-dependent manner. [file 9768713.f1.pdf]

Supplementary materials

Supplementary Table1. Primary and secondary antibodies

Primary antibodies				
Product name	Catalogue Number	Host species	Concentration	Supplier
cleaved caspase3	ab49822	Rabbit	1：500	Abcam
cleaved-caspase9	10380-1-AP	Rabbit	1：400	Proteintech
cytochrome C	10993-1-AP	Rabbit	1：750	Proteintech
Rab27a	ab55667	Mouse	1：1000	Abcam
HIF-1 α	20960-1-AP	Rabbit	1:750	Proteintech
BAX	50599-2-Ig	Rabbit	1：5000	Proteintech
BCL-2	60178-1-Ig	Mouse	1：4000	Proteintech
CD9	60232-1-Ig	Mouse	1：2000	Proteintech
CD63	25682-1-AP	Rabbit	1：500	Proteintech
CD81	ab109201	Rabbit	1：4000	Abcam
TSG101	14497-1-AP	Rabbit	1：2000	Proteintech
Calnexin	66903-1-Ig	Mouse	1：10000	Proteintech
β-actin	60008-1-Ig	Mouse	1：5000	Proteintech
Secondary antibodies				
HRP goat anti-mouse IgG	SA00001-1		1：5000	Proteintech
HRP goat anti-rabbit IgG	SA00001-2		1：6000	Proteintech

Supplementary Figure1

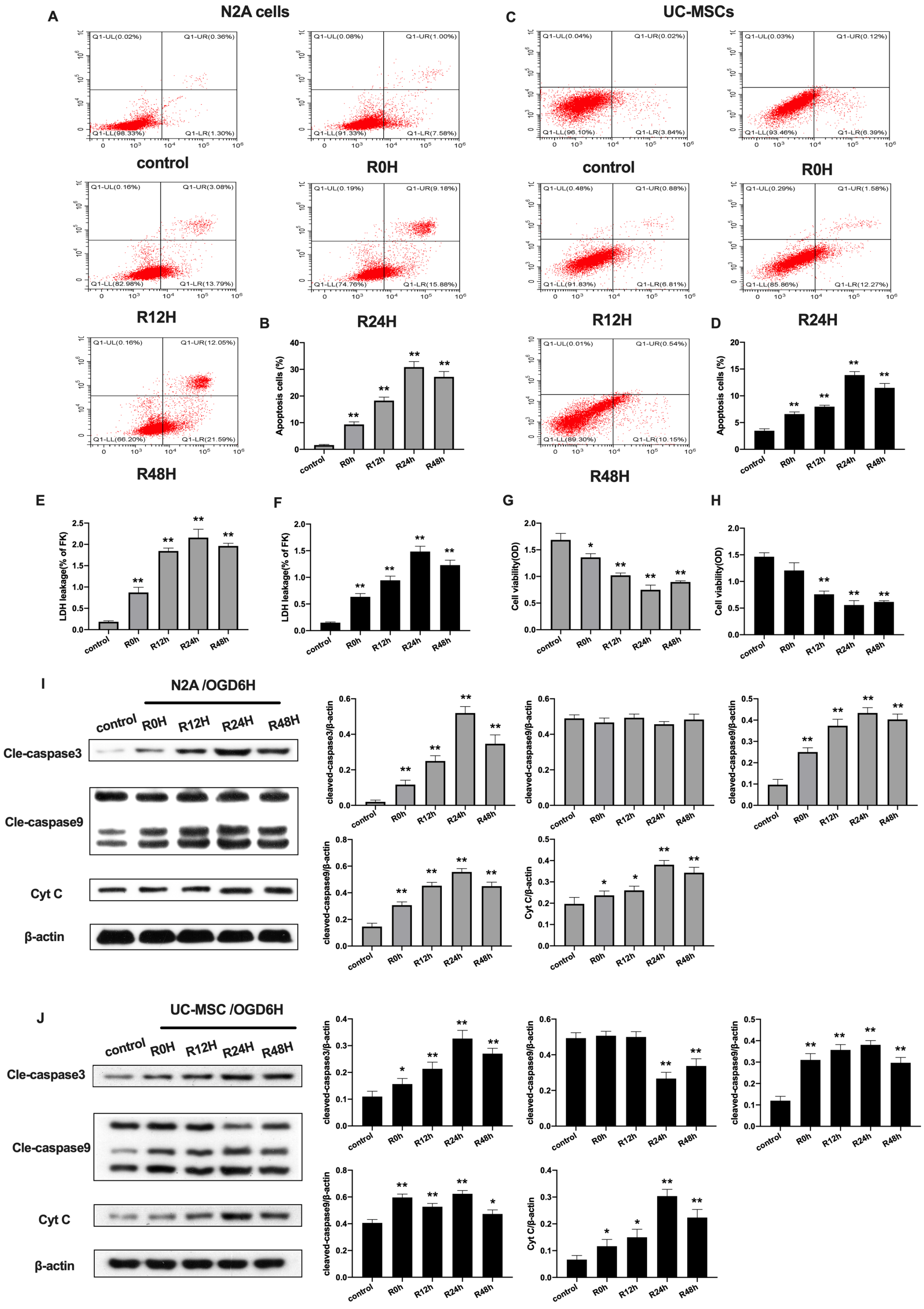

Supplementary Figure 1 OGD/R induced apoptosis of N2A cells and UC-MSCs during reperfusion in a time-dependent manner. N2A cells and UC-MSCs were subjected to OGD/R insult. (A-D) Apoptosis of N2A cells and UC-MSCs as evaluated by flow cytometry with Annexin-V/PI staining assay. (E-F) Apoptosis of N2A cells and UC-MSCs as evaluated by LDH leakage assay. (G-H) Cell viability of N2A cells and UC-MSCs as evaluated by MTT assay. (I-J) Expression

of cleaved-caspase3, cleaved-caspase9 and cytochrome C in N2A cells and UC-MSCs as evaluated by western blotting. All data are presented as the mean value ±SD(n=3).*p<0.05; **p<0.01, compared with control group.

Supplementary Figure2

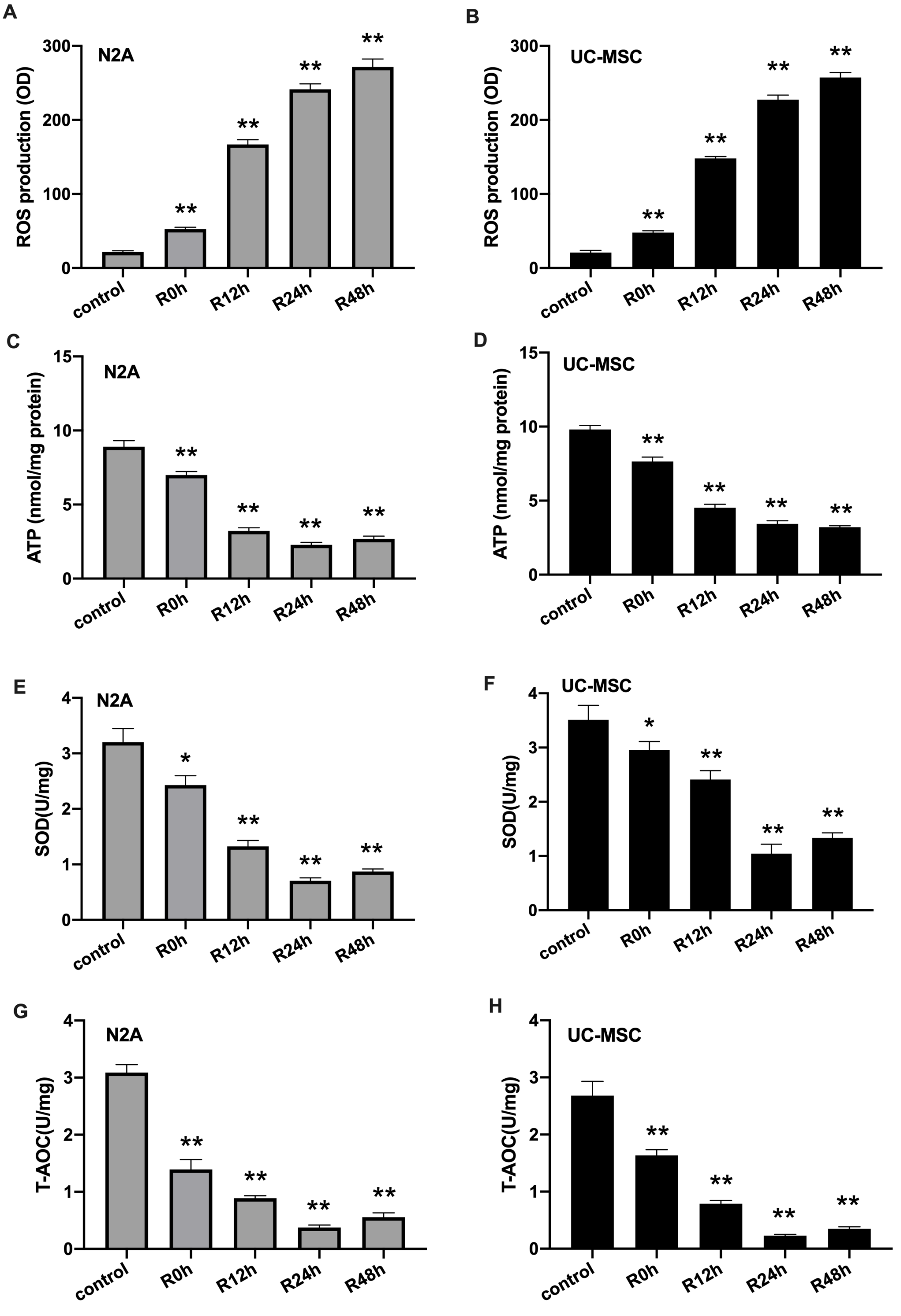

Supplementary Figure 2 OGD/R induced oxidative stress in N2A cells and UC-MSCs during

reperfusion in a time-dependent manner. N2A cells and UC-MSCs were subjected to OGD/R insult. (A-B) Levels of ROS in N2A cells and UC-MSCs as determined by dihydroethidium-DCFH-DA assay. (C-D) Levels of ATP in N2A cells and UC-MSCs as determined by firefly luciferase assay.(E-F) Levels of SOD in N2A cells and UC-MSCs as determined by WST-8 assay.（G-H） Levels of T-AOC in N2A cells and UC-MSCs as determined by ABTS assay. All data are presented as the mean value ±SD(n=3).*p<0.05; **p<0.01, compared with control group.
